# Embryoid Body Cells from Human Embryonic Stem Cells Overexpressing Dopaminergic Transcription Factors Survive and Initiate Neurogenesis via Neural Rosettes in the Substantia Nigra

**DOI:** 10.3390/brainsci13020329

**Published:** 2023-02-14

**Authors:** Rodrigo Ramos-Acevedo, Carmen Alejandra Morato-Torres, Francisco J. Padilla-Godínez, Luis Daniel Bernal-Conde, Marcela Palomero-Rivero, Faria Zafar, Omar Collazo-Navarrete, Luis O. Soto-Rojas, Birgitt Schüle, Magdalena Guerra-Crespo

**Affiliations:** 1 Department of Physiology, Faculty of Medicine, National Autonomous University of Mexico, Coyoacan, Mexico City 04510, Mexico; 2 Department of Molecular Neuropathology, Institute of Cell Physiology, National Autonomous University of Mexico, Coyoacan, Mexico City 04510, Mexico; 3 Department of Pathology, Stanford University School of Medicine, Stanford, CA 94305, USA; 4 National Laboratory of Genomic Resources, Institute of Biomedical Research, National Autonomous University of Mexico, Coyoacan, Mexico City 04510, Mexico; 5 Laboratory of Molecular Pathogenesis, Laboratory 4, Building A4, Medical Surgeon Career, Faculty of Higher Studies Iztacala, National Autonomous University of Mexico, Tlalnepantla 54090, State of Mexico, Mexico

**Keywords:** embryoid body cells, human embryonic stem cells, stem-cell transplantation, substantia nigra, neural rosettes, transcription factors

## Abstract

Transplantation of immature dopaminergic neurons or neural precursors derived from embryonic stem cells (ESCs) into the substantia nigra pars compacta (SNpc) is a potential therapeutic approach for functional restitution of the nigrostriatal pathway in Parkinson’s disease (PD). However, further studies are needed to understand the effects of the local microenvironment on the transplanted cells to improve survival and specific differentiation in situ. We have previously reported that the adult SNpc sustains a neurogenic microenvironment. Non-neuralized embryoid body cells (EBCs) from mouse ESCs (mESCs) overexpressing the dopaminergic transcription factor *Lmx1a* gave rise to many tyrosine hydroxylase (Th^+^) cells in the intact and damaged adult SNpc, although only for a short-term period. Here, we extended our study by transplanting EBCs from genetically engineered naive human ESC (hESC), overexpressing the dopaminergic transcription factors *LMX1A*, *FOXA2*, and *OTX2* (hESC-LFO), in the SNpc. Unexpectedly, no graft survival was observed in wild-type hESC EBCs transplants, whereas hESC-LFO EBCs showed viability in the SNpc. Interestingly, neural rosettes, a developmental hallmark of neuroepithelial tissue, emerged at 7- and 15-days post-transplantation (dpt) from the hESC-LFO EBCs. Neural rosettes expressed specification dopaminergic markers (Lmx1a, Otx2), which gave rise to several Th^+^ cells at 30 dpt. Our results suggest that the SNpc enables the robust initiation of neural differentiation of transplanted human EBCs prompted to differentiate toward the midbrain dopaminergic phenotype.

## 1. Introduction

Parkinson’s disease (PD) is a complex neurodegenerative disorder caused primarily by the loss of the mesencephalic dopaminergic neurons in the substantia nigra pars compacta (SNpc) that innervate the striatum [1,2]. The consequent depletion of dopamine in the striatum results in motor (bradykinesia, tremor, and difficulty initiating movement) and non-motor symptoms (cognitive changes, sleep disorders, depression, etc.) [3]. New strategies to slow disease progression or even replace lost cells are urgently needed, as current pharmacological treatments are only symptomatic and have long-term side effects [4,5].

Cell-based regenerative approaches are promising treatments, which historically include the engraftment of dopaminergic neurons from different cellular sources initially into the striatum [6,7] and later in the SNpc [8,9,10,11,12,13]. Recently, the interest in the SNpc has reemerged as it is the required niche to achieve the physiological regulation of mesencephalic dopaminergic neurons. Although this approach requires the specific axonal projections of the A9 DA subtype to the dorsolateral striatum, significant advances in restoring motor function have been demonstrated in the aforementioned studies.

In this regard, two types of pluripotent stem cells, embryonic stem cells (ESCs) and induced pluripotent stem cells (iPSCs), represent a reliable and reproducible cell source for transplantation in PD, both in the striatum and SNpc at different developmental stages. Human ESCs (hESCs) and human iPSCs (hiPSCs) have been transplanted as early as floor-plate progenitors at “late” stage, more advanced in the development as dopaminergic committed neuroblasts, immature dopaminergic neurons, or fully differentiated dopaminergic neurons, even though the latter are not actually considered as a therapeutical option [14]. Particularly, different groups have determined their efficiency in reconstructing the damaged nigrostriatal pathway [9,10,15]. Despite these remarkable advances, improving long-term survival and efficient differentiation in situ is a priority to regain a functionally regulated nigrostriatal circuit. Therefore, a thorough understanding of the adult SNpc niche will provide new insights for finding successful cell-therapy replacement strategies. In this regard, we have been studying the permissiveness of the SNpc to the neuronal and DA differentiation at the earliest stage of differentiation of ESC, by using embryoid body (EB) cells (EBCs) derived from mouse ESCs (mESCs) [16,17]. EBCs are cell aggregates that provide a well-studied model of embryonic development in vitro, expressing early embryonic markers in a compartmentalized manner [18,19]. Indeed, it has been demonstrated that EBCs derived from mESCs are responsive to instructive signals that direct them to differentiate progressively into mesencephalic neurons in the ventral midbrain niche at embryonic day 10.5 [20]. In addition, in this line of work, we reported that EBCs from wild-type mESCs can survive and differentiate into neurons in the adult rodent SNpc in vivo as efficiently as EBCs implanted in the rostral migratory stream, a recognized neurogenic niche; nonetheless, they were incapable of acquiring a dopaminergic cell fate [16]. Interestingly, we observed that the untreated adult SNpc was a permissive niche for dopaminergic differentiation of transplanted EBCs from mESCs overexpressing the murine LIM homeobox transcription factor 1 alpha (*Lmx1a*) (R1B5/NesE-Lmx1a cellular line), which has a fundamental role for the specification of the dopaminergic lineage during development [17]. Noteworthy, we also observed Th^+^ cells rising from EBCs from wild-type mESCs only in the 6-hydroxydopamine (6-OHDA)-lesioned SNpc, suggesting that the insult generates a local microenvironment promoting the dopaminergic phenotype.

In addition to *Lmx1a*, the transcription factors forkhead box A2 (*FoxA2*), and orthodenticle homeobox 2 (*Otx2*) are known to be essential for controlling the specification and maintaining the dopaminergic mesencephalic phenotype [21]. Furthermore, Zhu et al. demonstrated that simultaneous overexpression of human transcription factors *LMX1A, FOXA2*, and *OTX2* (herein collectively referred to as LFO) enhanced dopaminergic differentiation in vitro of EBCs from an hESC cell line (named hESC-LFO) [22]. The transcription activator-like effector nucleases (TALEN) engineered transgene cassette containing the transcription factors was under the transcriptional control of the nestin promoter to ensure its transcription during early neural differentiation in vitro [22].

Based on these in vitro results, we extended our previous studies to determine whether Th^+^ differentiation occurs in the intact and lesioned SNpc from wild-type hESC and the hESC-LFO line elicited to differentiate into dopaminergic neurons when transplanted at the EBC stage of differentiation. Interestingly, wild-type hESC EBCs were incompatible with cell survival, whereas hESC-LFO EBCs showed notable viability and differentiated into Lmx1a^+^, Otx2^+^, and doublecortin (Dcx)^+^ neural rosettes, an essential step in neuroepithelial cell development, at 7- and 15-days post-transplantation (dpt). Moreover, at 30 dpt, in a few animals, transplanted cells exhibited neuronal morphology, and in some areas of the graft an important number of Th-expressing cells was observed in the untreated and lesioned conditions. Thus, our results suggest that adult SNpc possesses the microenvironment to allow survival only of EBCs derived from hESC that overexpress midbrain LFO transcription factors. In addition, it is suggested that the adult SNpc is also permissive for the initiation of efficient neurogenesis, a knowledge that broadens the understanding of the limits of the regenerative capacity of the adult brain under physiological and pathological conditions.

## 2. Materials and Methods

### 2.1. Human Pluripotent Stem Cells Lines

For cell transplantation, we used the genetically engineered hESC-LFO (H9-LFO) line, and as controls the wild-type hESC (H9-PC) and wild-type hiPSC (PI-1761) developed by Zhu et al. [22]. The human pluripotent stem-cell lines used in this research were genetically modified from H9 hESC [23] using the dual integrase cassette exchange (DICE) strategy at the H11 safe harbor locus to insert a donor cassette for the transcription factors *LMX1A*, *FOXA2*, *OTX2*, and EGFP and puromycin resistance, and mCherry genes for selection and screening (Addgene, #51551). The expression of the donor cassette in the hESC-LFO can be tracked by the expression of the EGFP. The control cell lines H9-PC and PI-1761 only contain the puromycin resistance and mCherry genes.

### 2.2. Embryoid Body Culture

The unbiased neural differentiation protocol standardized by Zhu et al. was performed for EBs generation [22]. Briefly, hESCs were cultured on mouse-embryonic feeders for 7 days. Afterward, cell colonies were manually dissected and passaged onto feeder-free Geltrex (Thermo Fisher, Waltham, MA, USA)-coated plates with STEMFLEX medium (Thermo Fisher). The colonies were then cultured for 4 days. To form EBs, 1 mg/mL collagenase IV (Sigma-Aldrich, St. Louis, MO, USA) dissolved in E6 medium (Thermo Fisher) was added to the culture plate to detach the hESCs. The detached colonies were transferred to ultra-low adhesion culture wells (Sigma-Aldrich) and cultured for 4 days with E6 medium supplemented with 15% inactivated fetal bovine serum (Thermo Fisher). On the fourth day, EBs were rinsed twice with phosphate-buffered saline (PBS, Thermo Fisher) for transplantation. Dissociation of EBs was performed using a mixture of collagen IV (1 mg/mL), trypsin (0.05%, Gibco, Waltham, MA, USA), and EDTA (0.5 mM, Sigma) for 3 min, followed by manual pipetting to obtain small clumps. Subsequently, 3 mL of EB media (E6 medium with heat-inactivated fetal bovine serum) was added to inactivate the enzymatic mixture. Cells were centrifugated at 1000 rpm for 3 min and the supernatant was removed. Finally, cells were resuspended in 10 mL of PBS for transplantation [24].

Before transplantation and prior to dissociation of EBs, the formed EBs were monitored in vitro for activation of LFO transcription factors by EGFP expression, along with survival (by trypan blue) and morphological homogeneity as a quality control procedure.

### 2.3. 6-Hydroxydopamine Lesion and Cell Transplantation

Adult male Wistar rats (250–280 g) were housed under standard conditions: 12-h light/dark cycle, controlled room temperature (27 °C), and ad libitum access to food and water. Rats were obtained from the Institute’s animal facility. All procedures were conducted in accordance with the Guide for the Use and Care of Laboratory Animals of the National Institute of Health [25]. The protocol was approved by the Institutional Animal Care and Use Committee of the Institute of Cell Physiology (permit number: MGC65-19) of the National Autonomous University of Mexico. The number of animals was kept to a minimum and all efforts were made to reduce animal suffering. All methods are reported in concurrence with ARRIVE guidelines for describing animal experiments.

To generate a unilateral PD model, we used 6-OHDA, a neurotoxin that selectively and efficiently induce the death of the catecholaminergic neurons [26]. Prior to surgical procedures, animals were intraperitoneally anesthetized with a mixture of xylazine (8 mg/kg, PiSA, Mexico City, Mexico) and ketamine (100 mg/kg, PiSA), as we previously reported [17,27]. Anesthetized animals were mounted in a stereotaxic device and injected with a total volume of 0.5 µL of 0.9% of saline solution supplemented with 40 µg of 6-OHDA (Sigma-Aldrich) and 32 µg/µL of L-ascorbate (JT Baker, NJ, USA) into the left SNpc (coordinates: −4.7 mm AP, ±1.6 mm ML, −8.2 mm DV with respect to Bregma 0 [27,28]). A total of 127 rats were used in this study. The 6-OHDA infusion was injected into 64 animals at a rate of 0.125 mL/min [17]. The 6-OHDA group was compared with two control groups: 32 sham rats that were administered with 0.9% saline solution, and 31 untreated rats that underwent no treatment prior to cell transplantation (see Supplementary Table S1 for animal groups and the number of animals analyzed in each dpt).

For cell transplantation, animals were anesthetized as described above at 15 days after 6-OHDA infusion or 0.9% saline solution administration; this period of time was selected considering our previous studies where the lesion seems to be stabilized [16,17]. Subsequently, 3 µL of EBCs resuspended in PBS (described above in the culture section) containing approximately 60,000 cells were injected using a 5 µL-Hamilton syringe (Thomas Scientific, NJ, USA). Once the cell injection or 6-OHDA was completed, the cannula was left in place for at least 5 min to prevent back flow. To prevent graft rejection, cyclosporine A (10 mg/kg, PiSA) was orally administered daily until brain removal, initiating 1 day before cell transplantation.

### 2.4. Rotational Behavior

Two weeks after 6-OHDA administration, an amphetamine-induced rotation test was performed to confirm dopaminergic depletion in the nigrostriatal pathway (measure dopamine imbalance). Animals were injected intraperitoneally with amphetamine (4 mg/kg, Sigma-Aldrich). The number of left and right turns was recorded for 90 min using a custom-built computerized image and movement recognition system previously reported by our group [27]. Animals were evaluated 2 weeks after the 6-OHDA lesion, and only those with >400 ipsilateral turns were selected for transplantation procedures.

### 2.5. Histology Procedures

To analyze grafted EBCs at different developmental stages, rats were euthanized at 7, 15, or 30 dpt with sodium pentobarbital (60 mg/kg, PiSA) and intracardially perfused with 100 mL of 0.1 M phosphate buffer (JT Baker, NJ, USA), followed by 100 mL of ice-cold paraformaldehyde (4% *w/v* in 0.1 M PBS, PFA, Millipore, MA, USA). Brains were postfixed in 4% PFA for 12 h and cryoprotected by sequential 24-h incubations in 10%, 20%, and 30% sucrose solutions (in phosphate buffer, Millipore). Then, brains were sliced with a cryostat at 40 µm in the coronal plane and collected in an antifreeze solution.

Standard hematoxylin and eosin (H&E, Abcam, Cambridge, UK) staining procedures were performed to visualize cell morphology in the transplanted tissues [16]. For immunofluorescence, assays were performed, as we previously described [16,27]. Briefly, brain slices were treated three times with PBS/0.3% Triton X-100 and then with antigen retrieval citrate buffer (10 mM, pH 6.1, BioSB, Santa Barbara, CA, USA) at 65 °C for 30 min and washed three times with PBS. Subsequently, to avoid unspecific binding, brain slices were incubated for 1–2 h at room temperature in blocking solution composed of 0.1 M PBS, 0.3% Triton X-100 (Sigma-Aldrich), and 0.3% donkey serum (Gibco). The tissues were then incubated overnight at 4 °C with primary antibodies (listed in Supplementary Table S2) diluted in the blocking solution mentioned above. After rinsing 3 times in PBS, the slides were incubated for 2 h at room temperature with the corresponding Alexa Fluor secondary antibody (listed in Supplementary Table S2). Notably, the loss of the EGFP signal after fixation of the cells allowed us to use secondary antibodies at its corresponding wavelength. Finally, the sections were rinsed with PBS, and 4′,6-diamidino-2-phenylindole (DAPI; 1:10,000; Biostatus Limited, Shepshed, UK) was added to the final rinse for counterstaining of the cell nuclei. The human-specific antibody STEM121 (Takara Bio, Kusatsu, Japan) was used to distinguish between engrafted human and endogenous rat cells. Tissue was mounted on Superfrost slides (Thermo Fisher) with Black Diamond fluorescent mounting medium (Abcam).

Finally, to detect melanin aggregates, the Fontana-Masson staining kit (Sigma-Aldrich) was used according to the manufacturer’s instructions [29]. In short, slides were washed with distilled water, placed in 10% Fontana silver nitrate at 56 °C for 2 h, and rinsed 3 times in distilled water. The slides were then incubated in 1% gold chloride solution for 1 min and rinsed in distilled water. They were then placed in a 5% sodium thiosulfate solution for 1 min and rinsed thoroughly in distilled water twice. After, a standard staining with eosin solution was performed, and the sections were washed with distilled water and subsequently dehydrated with 50, 70, and 100% ethanol (Baker), followed by 100% xylene (Baker), and mounted as described in the previous section.

### 2.6. Image Acquisition and Cell Counting

For single-cell counting, images were captured on an Olympus FV1000 (Upright BX61WI and inverted IX81) confocal microscope. Photomicrographs of tissue transplants and neural rosettes were taken with a Zeiss Observer Z.1 spinning disk microscope and a Keyence BZ-X700 microscope. ImageJ/Fiji 1.53k software was used to quantify and classify neural rosettes and for 3D reconstructions. The 3D model of the transplant was made using a stack of ninety-four images.

### 2.7. Statistical Analysis

A one-way ANOVA was performed to compare the effect of the three SNpc conditions on the number of neural rosettes formed, at the two post-transplantation times. For statistically significant differences, a post hoc Tukey’s HSD test was performed to identify the significantly different groups. A 95% confidence interval was used for all statistical analysis.

## 3. Results

### 3.1. EBCs from hESC-LFO Cells Survive and Differentiate toward Neural Rosette Formation in the SNpc

**Figure 1:**
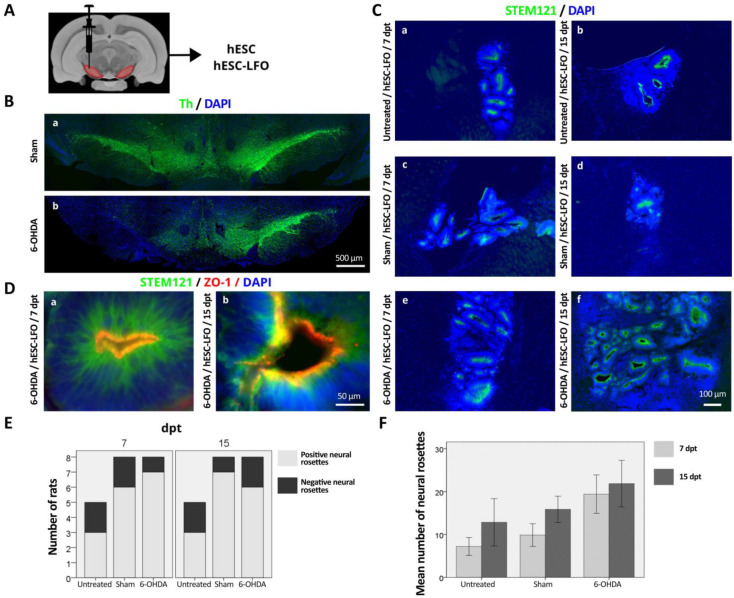
hESC-LFO EBCs differentiated into neural rosettes in the substantia nigra. (**A**) Schematic representation of EBCs transplantation protocol. EBCs from hESC and hESC-LFO cell lines were engrafted into SNpc, and at 7, 15, and 30 dpt brain tissue was analyzed. (**B**) Th expression in the SNpc of the (**a**) sham animals and the (**b**) 6-OHDA-lesioned rats 15 days after injury, at which time EBC engraftment takes place. (**C**) Representative photomicrographs of the neural rosettes found at (**a**,**c**,**e**) 7 and (**b**,**d**,**f**) 15 dpt in the SNpc of untreated (**a**,**b**), sham (**c**,**d**), and 6-OHDA-injured (**e**,**f**) rats. (**D**) Neural rosettes in the lesioned SNpc with STEM121 expression in green, and ZO-1 protein in red, expressed in the lumen of neural rosettes at (**a**) 7 and (**b**) 15 dpt. (**E**) Number of rats with grafts exhibiting neural rosettes (positive) or no neural rosettes (negative) according to the STEM121 human marker found in hESC-LFO transplants at 7 and 15 dpt on untreated (*n* = 5), sham (*n* = 8), and 6-OHDA (*n* = 8) conditions. (**F**) Mean number of neural rosettes present in untreated, sham, and 6-OHDA groups at 7 and 15 dpt. dpt: days post-transplantation; Th: tyrosine hydroxylase; ZO-1: zonula occludens 1.

The overexpression of *Lmx1a* in the mESC line R1B5/NesE-Lmx1a increases the mesencephalic DA differentiation capacity of EBCs transplanted in the untreated SNpc [17]. Here, to determine the potential of EBCs from hESC to differentiate into the SNpc, we transplanted EBCs from wild-type hESCs and the hESC-LFO line into this midbrain region. Our previous work demonstrated that brain damage caused by 6-OHDA generates a local environment that favors the survival of transplanted EBCs, and concomitantly promotes dopaminergic differentiation. Therefore, in this study, we compared the EBCs grafted cells in untreated SNpc, 6-OHDA injured SNpc, saline-injected (sham) SNpc of rats as a lesion control (Figure 1A–C,E,F). Surprisingly, immunofluorescence for STEM121 used to identify the human origin of the graft (not shown) and H&E staining, both at 7 and 15 dpt, showed that no grafts survived in the brains of animals transplanted with the EBCs derived from hESCs (see Supplementary Figure S1 and Supplementary Table S2, *n* = 41), or hiPSC EBCs used as an additional control of pluripotent stem-cell transplantation (see Supplementary Figure S2 and Supplementary Table S2, *n* = 26).

In contrast, the STEM121 signal showed robust survival in grafts from EBCs from hESC-LFO at 7 and 15 dpt (Figure 1C). Interestingly, the cellular morphology and STEM121 signaling showed that EBCs evolved into radial arrangements of columnar cells surrounding a central lumen that resembled the structure of neural rosettes in untreated, sham, and 6-OHDA-lesioned rats at 7 and 15 dpt (Figure 1C–E). Additionally, 100% of the surviving transplants at 7 and 15 dpt in the whole-time curse analyzed exhibited the homogeneous presence of neural rosettes (Supplementary Table S1).

The marker zonula occludens 1 (ZO-1) is expressed in the lumen of neural rosettes generated in vitro. Therefore, we used it as a bona fide marker for neural rosettes [30,31,32,33]. Indeed, ZO-1 was consistently expressed in the lumen of neural rosettes in 6-OHDA-lesioned rats at 7 and 15 dpt (Figure 1D), indicating that EBCs from hESC-LFO were able to differentiate into neural rosettes in the SNpc of adult rats. At 7 dpt, a one-way ANOVA revealed that there was a statistically significant difference in the mean number of neural rosettes between at least two SNpc conditions (F(2,14) = [3.764], *p* = 0.049). Nonetheless, Tukey’s HSD test for multiple comparisons found that there were no significant differences between groups, though with a clear tendency indicating that the 6-OHDA condition had more neural rosettes per graft than the sham or untreated conditions (Figure 1F). On the contrary, no significant differences were observed in the mean number of neural rosettes when comparing SNpc conditions at 15 dpt (F(2,17) = [0.934], *p* = 0.412) (Figure 1F), nor when comparing between same groups at different times: F(1,9) = [0.782], *p* = 0.399, untreated, F(1,12) = [2.205], *p* = 0.163, sham, and F(1,10) = [0.108], *p* = 0.750 (Figure 1F). These results suggest that the SNpc holds the capacity to allow EBCs from hESC-LFO to initiate neural differentiation via neural rosettes structure acquisition, in addition to the fact that a brain lesion raises local signals facilitating the neural differentiation [17].

### 3.2. Neural Rosettes Express Dopaminergic Lineage Markers in the Substantia Nigra

**Figure 2:**
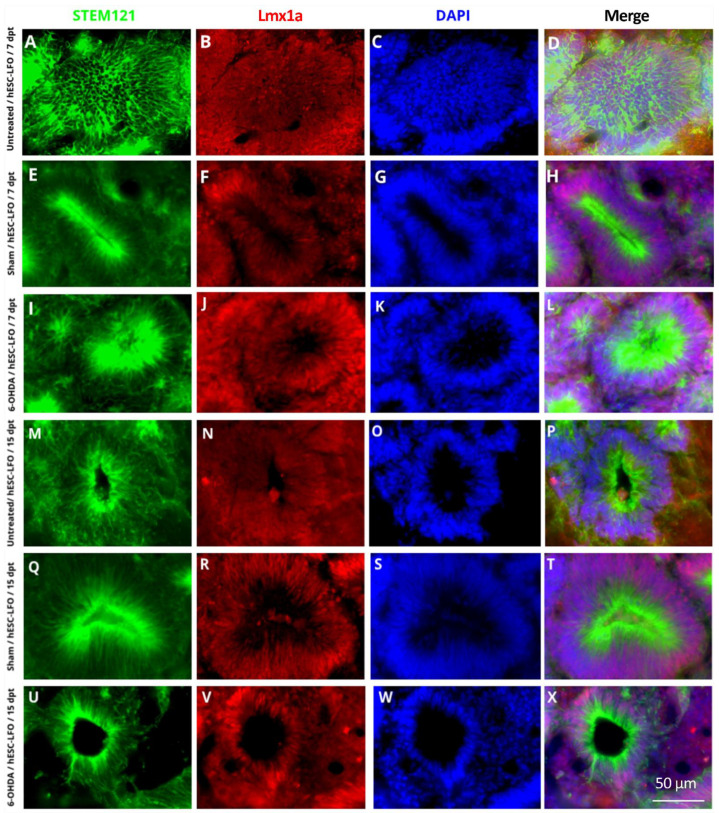
Neural rosettes of grafted hESC-LFO EBCs expressed Lmx1a at 7- and 15-days post-transplantation in the *substantia nigra*. Representative photomicrographs of neural rosettes from hESC-LFO EBCs grafts expressing Lmx1a. Neural rosettes cytoplasm was marked with the human cell antibody STEM121 (green). Lmx1a signal (red) was present in the nuclei of cells conforming the neural rosettes. DAPI was used to stain cell nuclei (blue). Transplanted tissue corresponds to (**A**–**D**,**M**–**P**) untreated, (**E**–**H**,**Q**–**T**) sham, and (**I**–**L**,**U**–**X**) 6-OHDA at (**A**–**L**) 7 and (**M**–**X**) 15 dpt. Lmx1a: LIM homeobox transcription factor 1 alpha; dpt: days post-transplantation.

**Figure 3:**
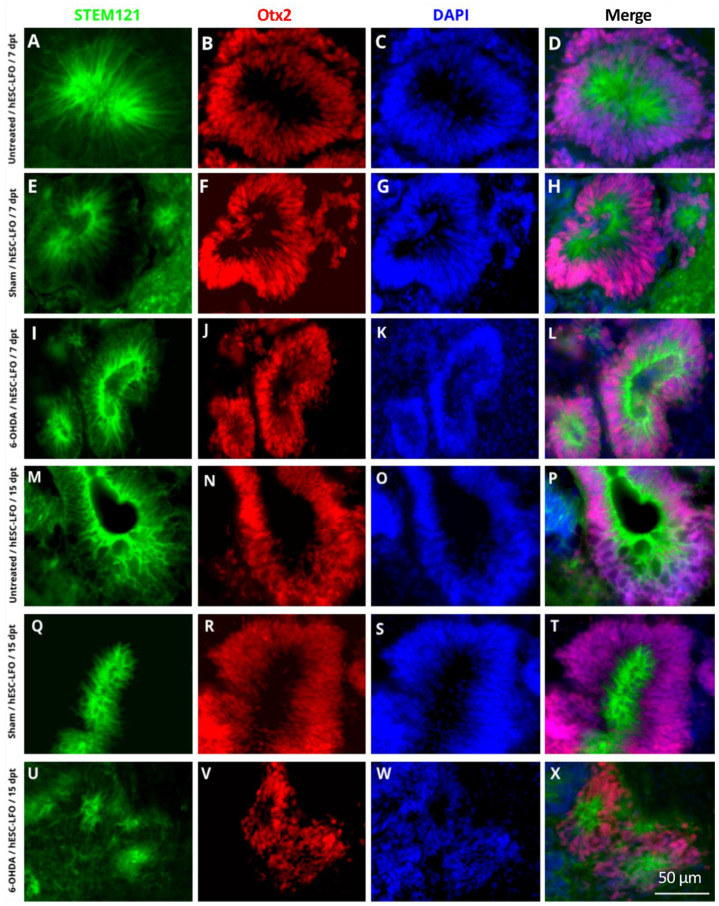
Neural rosettes of grafted hESC-LFO EBCs expressed Otx2 at 7- and 15-days post-transplantation in the *substantia nigra*. Representative micrographs of neural rosettes from the hESC-LFO EBCs grafts expressing Otx2. Neural rosettes cytoplasm was marked with the human cell antibody STEM121 (green). Otx2 signal (red) was also present in the cell nuclei of the neural rosettes. DAPI was used to stain cell nuclei (blue). Transplanted tissue corresponds to (**A**–**D**,**M**–**P**) untreated, (**E**–**H**,**Q**–**T**) sham, and (**I**–**L**,**U**–**X**) 6-OHDA at (**A**–**L**) 7 and (**M**–**X**) 15 dpt. dpt: days post-transplantation; Otx2: orthodenticle homeobox 2.

In ESCs, *Lmx1a* induces the mesencephalic specification throughout an autoregulatory loop with Wnt1; subsequently, Lmx1a-Wnt directly regulates Otx2, a downstream transcription factor also required for midbrain development [34]. Therefore, immunofluorescent assays were conducted to determine whether Lmx1a and Oxt2 transcription factors were expressed in the neural rosette formation. Indeed, Lmx1a (Figure 2) and Otx2 (Figure 3) were consistently observed in the nuclei of neural rosettes in STEM121-positive cells at 7 dpt (Figure 2A–L and Figure 3A–L) and 15 dpt (Figure 2M–X and Figure 3M–X) in untreated (Figure 2A–D,M–P and Figure 3A–D,M–P), sham (Figure 2E–H,Q–T and Figure 3E–H,Q–T), and 6-OHDA (Figure 2I–L,U–X and Figure 3I–L,U–X) rats. However, it was not possible to determine whether the expression of Lmx1a and Otx2 derived from the endogenous locus or the introduced transgene.

### 3.3. Neural Rosettes Are Mainly at the Differentiation Stage of Lumen Formation

**Figure 4:**
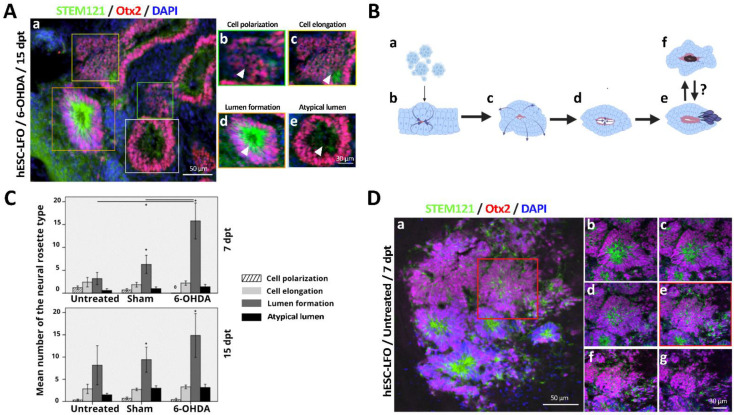
Neural rosettes of grafted hESC-LFO EBCs are mainly present in the lumen formation stage. (**A**) Representative image of different types of neural rosettes according to the STEM121-Otx2 labeling: (**a**) 20× transplantation image of the hESC-LFO EBCs into 6-OHDA-lesioned rat at 15 dpt with various types of neural rosettes; (**b**) cell polarization; (**c**) cell elongation; (**d**) lumen formation and (**e**) “atypical lumen”. (**B**) Graphical representation of neuronal differentiation of neural rosettes formed from hESC EBCs transplanted into the SNpc: (**a**) single cells and small clusters of EBCs are transplanted into the SNpc; (**b**) transplanted cells attach; (**c**) polarization, cells organize in an apical-basal conformation; (**d**) cell elongation, cells acquire an elongated morphology that allows the development of the lumen; (**e**) neural rosettes with lumen formation, a luminal part is formed, characterized by expression of the tight-junction marker ZO-1. The apical space begins to close, and cells migrate to the apical area; (**f**) unknown factors promote the generation of neural rosettes classified as atypical lumen, which present reduced STEM121 signal and small spaces devoid of cells in the lumen zone (white arrow). It is unknown whether (**e**) the lumen formation stage generates the (**f**) atypical lumen stage or vice versa. (**C**) Mean number of the neural rosettes type of untreated, sham, and 6-OHDA-lesioned rats at 7 and 15 dpt. Quantification of neural rosettes classification was performed using tissue marked with Otx2 colocalizing with STEM121. Error bars represent standard deviation. (*) indicates a significance of *p* < 0.05 calculated by one-way ANOVA. (**D**) Representative image of neural rosettes from an untreated rat at 7 dpt expressing Otx2 and STEM121. (**a**) 20× magnification of the hESC-LFO transplantation with neural rosettes. The red square highlights the area of (**b**–**g**) magnification. (**b**–**g**) Sliding levels from the top (**b**) to bottom (**g**) at 40× magnification of the red square in (**a**); 29 slides were captured with a width of 204.8 µm. (**e**) The image corresponds to the middle of the stack and is at the same slide level as the image in (**a**). Otx2: orthodenticle homeobox 2; dpt: days post-transplantation.

Next, we determined the differentiation stage of neural rosettes. Therefore, we categorized and quantified the neural rosettes found in untreated, sham, and 6-OHDA-lesioned rats at 7 and 15 dpt, according to the five stages of progressive morphological changes described in vitro by Hribková et al. It consists of cell intercalation, cell constriction, cell polarization, cell elongation, and the more mature stage, lumen formation (the lumen being essential for neural differentiation) [33]. Based on the cytoplasmatic signal given by the STEM121 human marker and the nuclear signals of Otx2 and DAPI, we observed that the neural rosettes present in our transplants were only at the stages of cell polarization, cell elongation, and lumen formation (Figure 4(Aa–d,Bc–e)). Additionally, we observed that a certain number of neural rosettes were morphologically different from the proposed in vitro classification and named them “atypical lumen” (Figure 4(Ae,Bf)). These neural rosettes were characterized by a lower STEM121 signal in the lumen zone, where one or more spaces with no cells were also observed (Figure 4(Aa,e)) [34].

At 7 dpt, we observed that there were significant differences between rosette stages in the untreated (F(3,9) = [5.479], *p* = 0.020) and the 6-OHDA (F(2,11) = [10.650], *p* = 0.003) groups, but not for the sham group (F(3,17) = [3.185], *p* = 0.051), according to ANOVA analysis (Figure 4C). The Tukey’s HSD tests determined that in the untreated and 6-OHDA groups the significantly different rosette stage was the lumen formation (Figure 4C). When comparing rosettes in the lumen formation stage between SNpc conditions, 6-OHDA-lesioned rats showed a significantly higher number of neural rosettes (F(2,12) = [4.086], *p* = 0.044) at the lumen formation stage (15.25 ± 3.16) than in sham (7.85 ± 1.71) or untreated rats (5.9 ± 2.48) (Figure 4C). Similarly, significant differences were also observed in the sham (F(3,21) = [4.978], *p* = 0.009) and 6-OHDA (F(3,17) = [6.315], *p* = 0.005) groups at 15 dpt (ANOVA), with the significantly different group being the lumen formation stage in both conditions, as determined by Tukey’s HSD tests (Figure 4C). These results indicate, according to our previous findings, that damaged SNpc induce a stronger neurogenic response from EBCs prompted to differentiate.

Additionally, we analyzed 3D stacks of neural rosettes (*n* = 5) in untreated SNpc to assess differences in morphology that could be given by the slide level obtained during 2D micrographs acquisition. We confirmed that most of the neural rosettes corresponded to the lumen formation stage, as the center of the neural rosettes showed a lumen surrounded by cells with elongated morphology (Figure 4D). Therefore, in the SNpc niche, the hESC-LFO EBCs underwent at least four morphological changes, indicative of their neural differentiation progress (Figure 4B).

**Figure 5:**
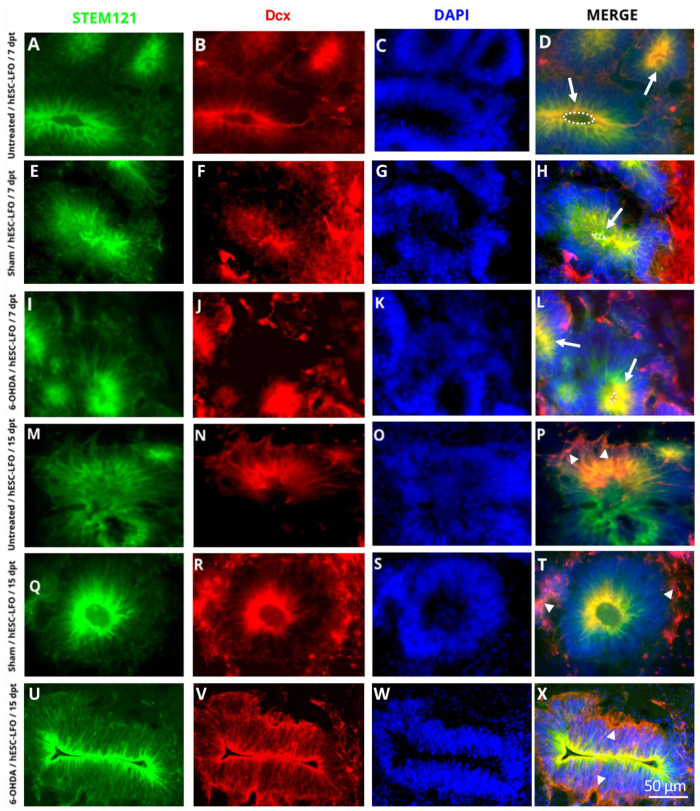
Neural rosettes of grafted hESC-LFO EBCs expressed Dcx at 7- and 15-days post-transplantation in the substantia nigra pars compacta. Representative images of neural rosettes from the hESC-LFO EBCs grafts expressing Dcx. Neural rosettes cytoplasm was marked with the human cell antibody STEM121 (green). Dcx signal (red) was mainly present in the lumen area of the neural rosettes at (**B**,**F**,**J**) 7 dpt, whereas at (**N**,**R**,**V**) 15 dpt it was more prevalent in the periphery of the neural rosettes. DAPI was used to stain cell nuclei (blue). Transplanted tissue corresponds to (**A**–**D**,**M**–**P**) untreated, (**E**–**H**,**Q**–**T**), sham, and (**I**–**L**,**U**–**X**) 6-OHDA at (**A**–**L**) 7 and (**M**–**X**) 15 dpt. White arrows in (**D**,**H**,**L**) indicate Dcx signals in the lumen of neural rosettes. Dotted ellipses in (**D**,**H**,**L**) indicate the apical zone. Arrow heads in (**P**,**T**,**X**) indicate Dcx signals surrounding the neural rosettes. dpt: days post-transplantation; Dcx: doublecortin.

**Figure 6:**
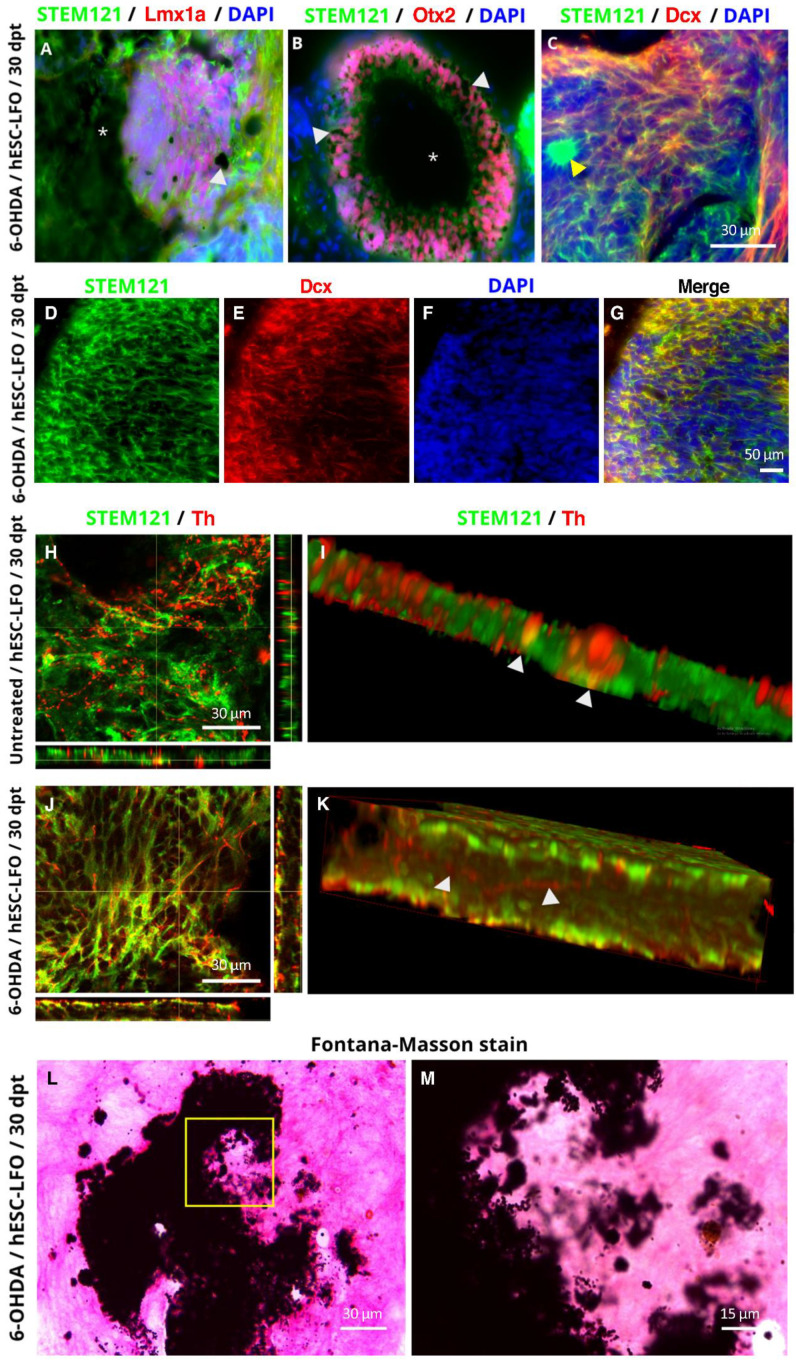
hESC-LFO EBCs grafts express tyrosine hydroxylase at 30 days post-transplantation in untreated and 6-hydroxydopamine-lesioned rats. (**A**–**C**) Representative images of the differentiation markers (**A**) Lmx1a, (**B**) Otx2, and (**C**) Dcx with STEM121 in hESC-LFO EBCs grafts at 30 dpt. Asterisks and white arrowheads indicate dark, pigmented areas with neuromelanin characteristics in (**A**,**B**), whereas the yellow arrowhead in (**C**) is showing a dense group of STEM121^+^ cells. (**D**–**G**) A high number of Dcx^+^ cells are colocalizing with the STEM121 human marker, as observed at a lower magnification (40×). (**H**–**K**) Representative photomicrographs of untreated (**H**,**I**), and 6-OHDA-lesioned conditions (**J**,**K**), with positive signals for Th and STEM121. (**H**) Orthogonal image of a double-labeled Th-STEM121^+^ cell in the untreated rat at 30 dpt. The white arrowheads indicate a double-labeled Th-STEM121^+^ soma. (**I**) Frontal view of a 3D reconstruction from (**H**). (**J**) Orthogonal image showing Th-STEM121^+^ cell in a lesioned rat at 30 dpt. The white arrowhead indicates a projection double labeled for Th and STEM121. (**K**) Lateral view of a 3D reconstruction from (**J**). DAPI signal is not shown for image clarity from (**H**) to (**K**). (**L**) Representative photomicrographs of Fontana-Masson staining in the hESC-LFO EBC grafted area and (**M**) is an amplification of the area shown in the yellow square in (**L**). dpt: days post-transplantation; Dcx: doublecortin; Lmx1a: LIM homeobox transcription factor 1 alpha; Otx2: orthodenticle homeobox 2; Th: tyrosine hydroxylase.

Additionally, to evaluate whether transplanted cells were also progressing towards neuronal differentiation through the neuroblast stage, we assessed the expression of doublecortin (Dcx), a protein involved in neural migration expressed in neuronal committed progenitors and neuroblasts [35]. Notably, Dcx was found to colocalize with STEM121 at 7 (Figure 5A–L) and 15 dpt (Figure 5M–X) in untreated (Figure 5A–D,M–P), sham (Figure 5E–H,Q–T), and 6-OHDA (Figure 5I–L,U–X) conditions. Interestingly, the signal was especially prominent in the lumen (apical zone) of the neural rosettes at 7 dpt in sham and 6-OHDA-lesioned conditions (Figure 5B,F,J). In contrast, at 15 dpt, Dcx expression in the 6-OHDA group, and to a lesser extent in the sham group, was predominantly observed surrounding the neural rosettes (basal zone) (Figure 5N,R,V) (for quantification refer to Supplementary Table S3). Therefore, a remarkable differentiation process of these structures was apparent in the 6-OHDA rats, as cells seemed to differentiate first at the lumen and, later, in the basal (outer) zone. The above is consistent with that observed at 30 dpt, where the graft no longer showed neural rosette formation (Figure 6C–G). Instead, cells appeared spindle-shaped and with projections, suggesting that SNpc is permissive to the progressive development of neural rosettes into migrating neuroblasts and probably immature neurons of EBCs from hESC-LFO (Figure 6C–G).

### 3.4. hESC-LFO EBCs Grafts Express Tyrosine Hydroxylase and Neuromelanin at 30 Days Post-Transplantation

Next, to investigate whether the neural rosettes were able to continue toward the differentiation progress in the SNpc, we analyzed the grafts at 30 dpt. We found that the grafts survived only in a few animals (Supplementary Table S1), in 1 of 11 6-OHDA-lesioned rats and 2 control rats (Figure 6), or 3 out of 13 (23.07%) rats, whereas sham rats showed no graft survival (data not shown). In the surviving tissue, we first assessed the expression of Lmx1a, Otx2, and Dcx to compare with the initial neural differentiation observed at the previous early stages analyzed. Interestingly, the colocalization of the STEM121 labeling with the different markers allowed us to determine that no neural rosettes were remaining (Figure 6A–G), and only small groups of cells positive for STEM121 signal were observed (Figure 6C). Contrarily, although some cells maintained expression of the floor-plate markers Lmx1a (Figure 6A), and Otx2 (Figure 6B), a robust expression of Dcx (Figure 6C–G), a marker of neuroblasts and young neurons, was evident in cells showing leading processes (Figure 6C–G). Therefore, we assessed whether these cells were expressing the dopaminergic marker Th. Notably, all the brains exhibiting surviving transplants showed positive immunolabeling for Th, although not uniformly throughout the graft, but in groups of cells where not the total of cells were labeled (Figure 6H–K). The morphology of the engrafted cells visualized in a 3D reconstruction (Figure 6I,K) also showed Th- and STEM121-labeled neuronal projections colocalizing in the graft. This highlighted that quantification of Th^+^ cells were not possible due to the high density of cell bodies and their projections and the Th-stippled pattern exhibited by cells in the untreated SNpc, which does not allow adequate delineation of the projections (Figure 6H–K). However, it is possible to observe in cells transplanted from the lesioned SNpc a significant number of cells in which Th-STEM121 signals were colocalizing (Figure 6J,K), in contrast to cells transplanted in the untreated SNpc, where fewer labeled Th^+^ cells were detected (Figure 6H,I).

Furthermore, we observed that brain samples exhibiting cell survival, besides Th^+^ cells, also had a dark pigmentation resembling neuromelanin (Figure 6A,B), a bioproduct of dopamine oxidation in SNpc neurons [36]. A Fontana-Masson neuromelanin staining [37,38] confirmed its presence (Figure 6L,M). Therefore, at 30 dpt, grafted cells survived and differentiated into neuronal-like cells with emerging projections, and groups of cells were expressing Th and neuromelanin.

## 4. Discussion

Transplantation in the SNpc of PD patients pursues the integral and regulated reestablishment of the host nigrostriatal circuit, which could bring important advantages over current treatments. In animal models, the engraftment of pluripotent stem cells in the neural stem cell or differentiated stage have proven feasible to reach this goal [14]. Functional integration implies that the transplanted cells need to be able to survive, efficiently reach dopaminergic maturation, establish synapses with target neurons, and release dopamine under physiological conditions. Despite the promising results, it remains partially unanswered whether the SNpc holds the permissive or inductive microenvironment to allow fully a successful cell-based therapy.

We have previously shown that the intact adult SNpc is a neurogenic niche for the effective neuronal differentiation of primed mESC EBCs [16]. We also reported that the intact SNpc allowed mESC EBCs carrying Lmx1a to derive into dopaminergic neurons in the short term, indicating that the SNpc did not hamper the dopaminergic differentiation of EBCs prompted to differentiate into dopaminergic neurons [17]. In this work, we widened our previous studies to determine whether naive human EBCs grafted in the intact and damaged SNpc are capable of initiating neurogenesis and differentiating into Th^+^ cells per se, or if they require the addition of intrinsic factors such as Lmx1a FoxA2 and Otx2. Accordingly, we used a hESC line overexpressing the mentioned transcription factors that have already been demonstrated to enhance the differentiation of EBCs into dopaminergic neurons in vitro [22].

In this study, we found that the intact and 6-OHDA-lesioned adult SNpc is a niche with the capacity to enable survival only from hESC EBCs with the expression of the exogenous transcription factors LFO. Additionally, the hESC-LFO EBCs generated Lmx1a and Otx2 positive neural rosettes expressing also Dcx, therefore confirming a neuroblast stage. In this regard, it has been demonstrated that neurally uncommitted mouse EBCs are endowed with the ability to follow specification and neuralization cues of the embryonic midbrain that allows them to differentiate into dopaminergic neurons [20]. On the other hand, we determined that mouse EBCs have the potential to respond to environmental signals in the postnatal and adult rat brain, where they neuralize and progressively give rise to neurons [16]. Whereas in the striatum a small number of neurons were observed concomitantly with glial cells, in the SNpc a considerable number of neurons and non-glial cells were found, even though non-dopaminergic neurons arise from mouse EBCs [16] Interestingly, the forced expression of *Lmx1a* in the mouse EBCs derives into efficient dopaminergic specification when grafted in the SNpc, even though in the 6-OHDA-lesioned SNpc neuroblasts did not survive at 15 dpt [17]. Here, hESC EBCs overexpressing the LFO transcription factors are able to survive and reach an effective neuralization at 7 and 15 dpt, with the subsequent arousal of Th^+^ cells at 30 dpt both in the intact and lesioned SNpc.

The expression of the LFO transcription factors is necessary for the specification and maintenance of the dopaminergic mesencephalic phenotype in development and adulthood [21,39,40,41], and in vitro [22], as they regulate several genes with a critical role in the mesencephalic dopaminergic lineage, such as Pitx3, Girk2, or Nurr1 [21]. Here, LFO transcription factors were necessary for graft survival. However, the complete absence of survival of two wild-type pluripotent stem cells prevents us from defining whether the LFO transcription factors cooperatively contribute with the microenvironment to induce specification in vivo or, on the contrary, whether it is the SNpc that holds all the cues to follow specific differentiation; the latter seems unlikely based on our previous findings [16,17]. Nonetheless, our results underline the relevance of the LFO transcription factors to circumvent one of the main barriers of the PD transplantation approach.

We already demonstrated that mESC EBCs follow a strong neuronal differentiation upon middle cerebral artery occlusion injury [16]. Additionally, wild-type mESC EBCs in the 6-OHDA-lesioned SNpc give rise to dopaminergic neuroblasts, at a similar level of mESC EBCs expressing *Lmx1a* [17], suggesting the damage induces a state that promotes dopaminergic specification. Here, the formation of neural rosettes in our grafts indicated the development of neuroepithelial tissue, with a trend for a higher prevalence of neural rosettes and a significant increase of the lumen-forming stage in the injured SNpc compared with untreated SNpc. To our knowledge, this is the first report of differentiation via neural rosette formation in SNpc after transplantation of EBCs from hESCs. Moreover, we observed a neural rosette formation that we called “atypical” since was not reported in cell culture by Hribková et al., but a similar neural rosette cytoarchitecture with a central hollow was reported from hESC growth in micropatterned array cell culture [42]. However, the analysis performed so far is insufficient to locate this state temporally with respect to the formation of the lumen, so an experimental strategy to identify the progression of a single neural rosette over time would be necessary to determine whether this form precedes or proceeds it. Importantly, the extrinsic and possible intrinsic signals having a role in neural rosette formation have yet to be determined.

Furthermore, in the most advanced differentiated stage studied (30 dpt), in addition to Lmx1a, Otx2, and Th expression, the neuromelanin pigment deposits found in the grafted area were a direct indication of dopamine oxidation, suggesting that dopamine synthesis by the grafted cells is taking place, which is an indispensable feature of dopaminergic neurons that has also been observed in organoids directed towards a dopaminergic phenotype [43,44].

Despite the efficient neuralization found in the present study, the Th signal observed across the graft only in patched areas suggests that EBs probably did not effectively reach the stage of committed dopaminergic neural progenitors. The above could be happening since it has been shown that committed dopaminergic neural progenitor cells generated in vitro from hESCs when transplanted in the SNpc are able to efficiently differentiate into dopaminergic neurons that project axons to reinnervate the striatum and improve motor function [8,45,46].

It is essential to highlight that undifferentiated ESC grafts can generate non-cancerous overgrowths that may be detrimental to brain function [47]. Nonetheless, we did not observe tumorigenic growths at the intervals of time analyzed for EBC transplantation. On the contrary, the morphology development from neural rosettes towards individual cells, and the positive labeling to Dcx and Th with leading processes, suggest that the cells were in a progressive process of differentiation.

Instead of cellular overgrowth, the survival of the grafted cells decreased considerably as the transplantation time progressed. Only 23.07% of the rats at 30 dpt showed surviving cells, in contrast with the 74% at 7 and 15 dpt. At the same time, this survival difference could mean that more potent immunosuppression is needed to overcome possible immune reactions that promote the death of transplanted cells, as the rats used in this study were pharmacologically immunosuppressed. A genetic immunodeficient rat model or a combination of immunosuppressors, could improve cell survival and allow us a deeper study of the hESC-LFO EBCs differentiation in the SNpc in the longer term [48].

Here, our data show that the SNpc is a permissive microenvironment for efficient development of neuralization from hESC EBCs overexpressing specific dopaminergic transcription factors, but, in contrast, terminal differentiation is less strongly generated. It remains to be determined which intrinsic and/or extrinsic signals are required for long-term survival. At the same time, the signaling proteins present in the SNpc microenvironment that could be involved in maintaining the expression of LFO transcription factors are unknown. Several growth factors present in this niche have been demonstrated to have a role in survival or dopaminergic phenotype maintaining. The potential candidates are brain-derived neurotrophic factor (BDNF) [49,50,51,52,53,54], glial cell-derived neurotrophic factor (GDNF) [53,54,55,56,57,58,59], and angiogenic factors such as vascular endothelial growth factor (VEGF) [60,61,62]. Additionally, whether the immune response to engrafted cells is involved in preventing survival and, consequently, reaching a greater number of Th^+^ cells continue to be determined.

## 5. Conclusions

This study provides evidence that intact and injured SNpc promote survival and support neuralization of hESC-LFO EBCs. Thus, our results contribute to elucidate the potential of the SNpc to enable differentiation of genetically engineered hESC transplanted at a very early stage of development, which would mean that the SNpc harbor a greater capacity to allow survival and neuralization than previously reported. However, it is essential to unravel the microenvironmental cues present in the SNpc that contribute to drive survival and neural specification in vivo. On the other hand, our study suggests that the plasticity of non-neuralized cells (EBCs) from hESCs and probably hESCs-derived committed dopaminergic neural progenitor cells can be modified with transcription factors, and therefore, optimization of survival and dopaminergic differentiation in the SNpc can be highly improved in cell-replacement therapies.

## Data Availability

All data generated and analyzed during the current study are available from the corresponding author on reasonable request.

## Supplementary Materials

The following supporting information can be downloaded at: https://www.mdpi.com/article/10.3390/brainsci13020329/s1, Figure S1: Transplanted wild-type hESC EBCs did not survive in the *substantia nigra*; Figure S2: Transplanted wild-type hiPSCs did not survive in the *substantia nigra*; Table S1: Number of rats grafted and percentage of rats with surviving transplants; Table S2: List of antibodies used in this study; Table S3: Dcx distribution per neural rosettes at 7 and 15 dpt.

## Abbreviations

6-OHDA: 6-hydroxydopamine; Dcx, doublecortin; dpt, days post-transplantation; EB, embryoid body; EBCs, embryoid body cells; FoxA2, forkhead box A2; hESCs, human embryonic stem cells; H&E, hematoxylin and eosin; hESC-LFO, human embryonic stem cells overexpressing transcription factors Lmx1a, FoxA2, and Otx2; Lmx1a, LIM homeobox transcription factor; mESCs, mouse embryonic stem cells; Otx2, orthodenticle homeobox; PD, Parkinson’s disease; PFA, paraformaldehyde; SNpc, *substantia nigra pars compacta*; Th, tyrosine hydroxylase; ZO-1, zonula occludens.
