# Supplementary material for: Embryoid Body Cells from Human Embryonic Stem Cells Overexpressing Dopaminergic Transcription Factors Survive and Initiate Neurogenesis via Neural Rosettes in the Substantia Nigra
Source: Brain Sci. 2023 Feb 14;13(2):329. doi: 10.3390/brainsci13020329 (PMC9954545; doi:10.3390/brainsci13020329)
Supplement: Supplementary file 1 [file brainsci-13-00329-s001.zip › Figure S1.pdf]

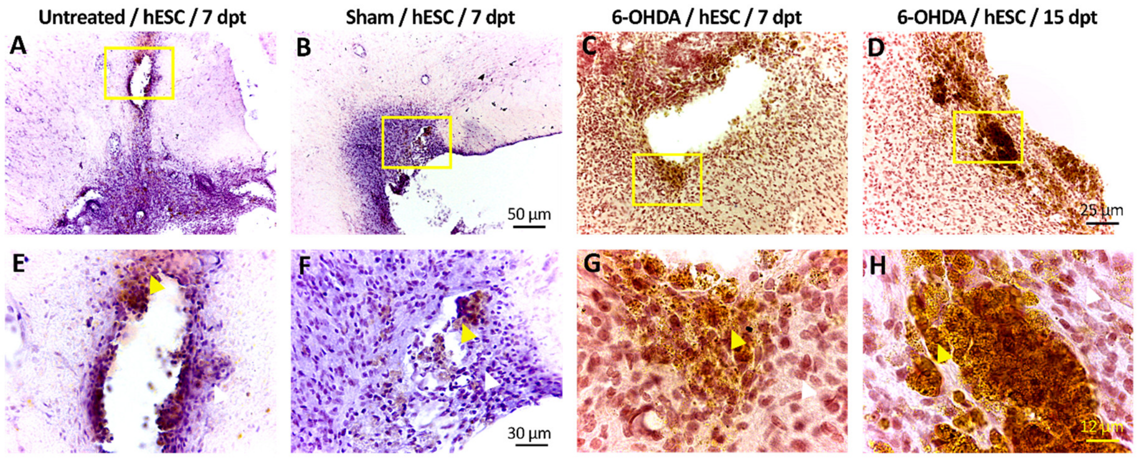

Figure S1. Transplanted wild-type hESC EBCs did not survive in the *substantia nigra*. Representative photomicroographs of H&E-stained brain slices from **A, E** Untreated at 7 dpt, **B, F** Sham at 7 dpt, **C, G, D, H** 6-OHDA-lesioned animals at 7 dpt (**C, G**) and 15 dpt (**D, H**) transplanted with wild-type hESC EBCs. **A, B, C, D** show the grafted area with cavitations surrounded by compacted cell clusters and a yellow pigment, characteristic of cell debris in H&E. The yellow rectangles in **A**, **B, C**, **D** represent the enlargement zones in **E**, **F**, **G**, **H** respectively. **E**, **F, G**, **H** allow us to appreciate the morphology of the cells, the white arrowheads point to the undamaged morphology of endogenous cells, whereas the yellow arrowheads indicate compacted cell clusters and damaged cells with aberrant nuclear morphology. A yellow pigment, characteristic of cell death, is observed in this same area. Magnifications for **A, B** are 10x; for **E, F,** are 40x; for **C, D** are 20x; and for **G, H** are 100x. dpt: days post-transplantation; EBCs: embryoid body cells; hESC: human embryonic stem cells; 6-OHDA: 6-hydroxydopamine.
