# Supplementary material for: Embryoid Body Cells from Human Embryonic Stem Cells Overexpressing Dopaminergic Transcription Factors Survive and Initiate Neurogenesis via Neural Rosettes in the Substantia Nigra
Source: Brain Sci. 2023 Feb 14;13(2):329. doi: 10.3390/brainsci13020329 (PMC9954545; doi:10.3390/brainsci13020329)
Supplement: Supplementary file 1 [file brainsci-13-00329-s001.zip › Figure S2.pdf]

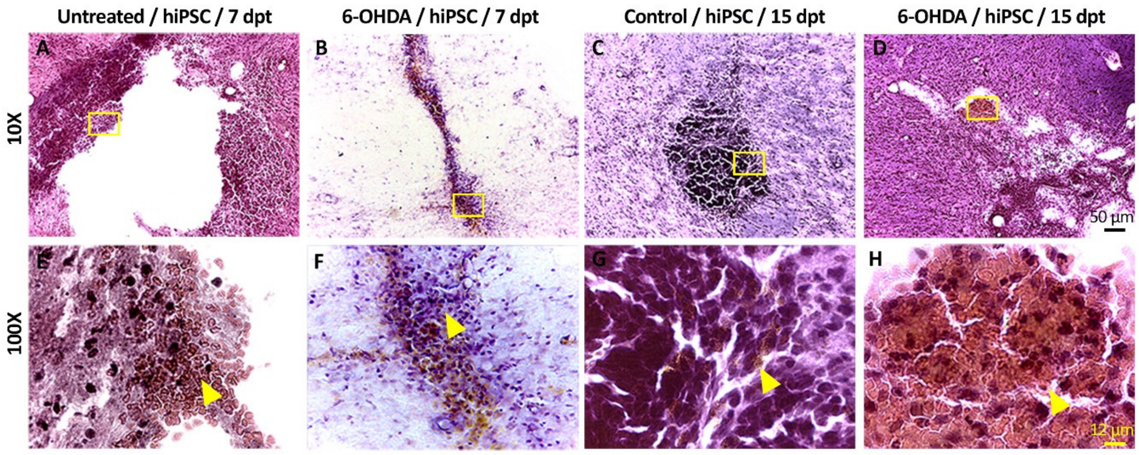

Figure S2. Transplanted wild-type hiPSCs did not survive in the *substantia nigra*. Representative images of H&E-stained brain slices at 7 dpt from **A, E** Untreated, and **B, F** 6-OHDA-lesioned animals, and at 15 dpt from **C, G** Untreated, and **D, H** 6-OHDA lesioned animal transplanted with hiPSC EBCs. **A, B, C, D** show cavitations in the grafted area surrounded by compacted cell clusters. The yellow rectangles in **A, B, C, D** represent the enlargement zones in **E, F, G, H**, respectively. In **A, B, C, D** the yellow arrowheads point to compacted cell clusters and damaged cells with aberrant nuclear morphology. A yellow pigment, characteristic of cell death, is observed in this same area. Magnifications for **A, B, C, D** are 10x, and for **E, F, G, H** are 100x. dpt: days post-transplantation; EBCs: embryoid body cells; hiPSCs: human induced pluripotent stem cells; 6-OHDA: 6-hydroxydopamine.
