# Supplementary material for: Embryoid Body Cells from Human Embryonic Stem Cells Overexpressing Dopaminergic Transcription Factors Survive and Initiate Neurogenesis via Neural Rosettes in the Substantia Nigra
Source: Brain Sci. 2023 Feb 14;13(2):329. doi: 10.3390/brainsci13020329 (PMC9954545; doi:10.3390/brainsci13020329)
Supplement: Supplementary file 1 [file brainsci-13-00329-s001.zip › Table S1.pdf]

Table S1. Number of rats grafted and percentage of rats with surviving transplants.

		hESC	hiPSC	hESC-LFO	
Condition	Dpt	Survival %	Survival %	Survival %	Presence of neural rosettes
Untreated	7	0% (n=3)	0% (n=5)	60% (n=5)	(+)
	15	0% (n=3)	0% (n=5)	60% (n=5)	(+)
	30	0% (n=3)	ND	100% (n=2)	(-)
Sham	7	0% (n=3)	ND	75 % (n=8)	(+)
	15	0% (n=5)	ND	87.5% (n=8)	(+)
	30	0% (n=3)	ND	0% (n=5)	(-)
6-OHDA	7	0% (n=8)	0% (n=8)	87.5% (n=8)	(+)
	15	0% (n=8)	0% (n=8)	75% (n=8)	(+)
	30	0% (n=5)	ND	9.09% (n=11)	(-)

dpt: days post-transplantation; ND: not done; Survival % (percentage rats with graft survival of total transplanted rats), n=total number of transplanted rats.
