# Supplementary material for: Embryoid Body Cells from Human Embryonic Stem Cells Overexpressing Dopaminergic Transcription Factors Survive and Initiate Neurogenesis via Neural Rosettes in the Substantia Nigra
Source: Brain Sci. 2023 Feb 14;13(2):329. doi: 10.3390/brainsci13020329 (PMC9954545; doi:10.3390/brainsci13020329)
Supplement: Supplementary file 1 [file brainsci-13-00329-s001.zip › Table S2.pdf]

Table S2. List of antibodies used in this study

Antibody	Type	Dilution	Company
Zonula occludens 1 (ZO-1)	Primary	1:100	BD
STEM121	Primary	1:1000	Millipore
Lmx1a	Primary	1:1000	Millipore
Otx2	Primary	1:1000	Thermo Fisher
Doublecortin (Dcx)	Primary	1:1000	Millipore
Tyrosine hydroxylase (Th)	Primary	1:500	Thermo Fisher
Alexa Fluor 458 Anti-Mouse	Secondary	1:1000	Millipore
Alexa Fluor 645 Anti-Goat	Secondary	1:400	Millipore
