# Supplementary material for: Embryoid Body Cells from Human Embryonic Stem Cells Overexpressing Dopaminergic Transcription Factors Survive and Initiate Neurogenesis via Neural Rosettes in the Substantia Nigra
Source: Brain Sci. 2023 Feb 14;13(2):329. doi: 10.3390/brainsci13020329 (PMC9954545; doi:10.3390/brainsci13020329)
Supplement: Supplementary file 1 [file brainsci-13-00329-s001.zip › Table S3.pdf]

Table S3. Dcx distribution *per* neural rosettes at 7 and 15 dpt.

	7 dpt		15 dpt	
	**% DCX in apical zone (lumen)**	**% DCX in basal zone**	**% DCX in apical zone (lumen)**	**% DCX in basal zone**
Sham	45.45 ± 6.43	0.00 ± 0.00	9.30 ± 10.40	11.63 ± 11.03
6-OHDA	47.37 ± 39.74	2.63 ± 3.72	21.67 ± 20.82	25.00 ± 11.79

The percentage was obtained from the quantification of the Dcx distribution by individual neural rosettes in at least two slices, in three different areas, from 2 rat brains. dpt: days post-transplantation.
